# Extracting reaction networks from databases–opening Pandora’s box

**DOI:** 10.1093/bib/bbt058

**Published:** 2013-08-14

**Authors:** Liam G. Fearnley, Melissa J. Davis, Mark A. Ragan, Lars K. Nielsen

**Keywords:** databases, signal transduction, modelling, reaction networks

## Abstract

Large quantities of information describing the mechanisms of biological pathways continue to be collected in publicly available databases. At the same time, experiments have increased in scale, and biologists increasingly use pathways defined in online databases to interpret the results of experiments and generate hypotheses. Emerging computational techniques that exploit the rich biological information captured in reaction systems require formal standardized descriptions of pathways to extract these reaction networks and avoid the alternative: time-consuming and largely manual literature-based network reconstruction. Here, we systematically evaluate the effects of commonly used knowledge representations on the seemingly simple task of extracting a reaction network describing signal transduction from a pathway database. We show that this process is in fact surprisingly difficult, and the pathway representations adopted by various knowledge bases have dramatic consequences for reaction network extraction, connectivity, capture of pathway crosstalk and in the modelling of cell–cell interactions. Researchers constructing computational models built from automatically extracted reaction networks must therefore consider the issues we outline in this review to maximize the value of existing pathway knowledge.

## INTRODUCTION

The first simulation of an entire cellular system [1] represents a watershed moment in computational biology. This simulation combines multiple models of cellular processes into a simulation capable of predicting the phenotype of the single-celled organism *Mycobacterium genitalium*, one of the simplest living organisms [2]. Because of this simplicity, the component process models are relatively small and can be manually assembled from the literature in a reasonable timeframe. However, this model assembly step presents a major hurdle to whole-cell systems modelling in more-complex organisms. Some processes, such as metabolism, have existing large-scale models [3]; others, such as the signal transduction system, do not. The simplest way to accelerate the development of large-scale models is to exploit data contained in existing knowledge bases. In the case of signal transduction, these databases are already used to interpret the results of high-throughput experiments, and provide detailed insight into the specific mechanisms through which phenotype is controlled and created. Open-access databases range in size and scope from small single-pathway models with extensive kinetic detail (e.g. those captured in the BioModels [4] database) to much larger, community-curated reaction databases such as Reactome [5, 6], the National Cancer Institute Pathway Information Database (PID) [7] and the Kyoto Encyclopedia of Genes and Genomes (KEGG) [8, 9]. While a number of commercial pathway knowledge bases are available for performing traditional pathway analysis [10], the costs, format, data sharing restrictions, and terms of use make these less attractive as a source of data for network extraction. Open-access databases are thus the main repositories of the large-scale pathway reaction and interaction data necessary for cellular-scale systems modelling.

Knowledge bases are generated by teams of skilled researchers and curators working on functionally defined pathways, entering data extracted from the body of literature relevant to that reaction system. These data are then checked by independent curators to ensure accuracy, and later revisited and updated to reflect changes in our understanding of the system. Such databases have three key features: (i) they are detailed, (ii) they undergo quality control processes and (iii) they are broad, covering many pathways in depth and detail (Reactome describes 12 334 participants in 6004 reactions across 1371 pathways in their *Homo sapiens* data set as of January, 2013). They also provide high-quality diagrammatic representations of their constituent pathways for visual interrogation and analysis.

Computational analyses of pathway systems that use these data range in sophistication from the use of pathway enrichment and gene-set enrichment techniques [11], through to simulations at the reaction level in systems ranging in size from single signal transduction pathways [12, 13] to genome-scale metabolic networks [14, 15], cellular-scale signalling networks [16] and whole-cell models [1].There are many and important distinctions among these simulation-based modelling and analytical techniques, but those more sophisticated than simple enrichment analyses require the extraction and use of the underlying reaction network, typically in the form of an adjacency matrix or adjacency list. This adjacency list is the most basic representation of a set of reactions possible, and is used (with additional information) to generate rate equations, flux-balance equations or Boolean statements [12, 13, 16, 17].

A number of design decisions in the implementation of pathway databases can hinder the extraction of this fundamental information. Previous discussions of the knowledge contained within databases have focused on the content of databases, specifically the completeness and quality of annotation in signal transduction [18] and metabolic [19] networks, rather than the impact and implications of these implementation choices for modelling and simulation. These features have not been widely discussed, and have been overlooked in many modelling applications. Other issues arise from the age of many of the current generation of database projects. Major databases take time to design, build and populate with curated data, and many major repositories have been running for 6 (PID) to 8 (KEGG, Reactome) years. Our understanding of and ability to detect certain biological phenomena has improved during this time. Early design decisions and subsequent shifts in knowledge have significant repercussions for modellers, database designers, curators and the broader community that rely on these analytical tools.

We will discuss issues related to knowledge representation, and to the capture of underlying biological complexity, from the perspective of computational pathway analysis. These problems are found across the range of currently available databases; we will provide representative examples from the Panther Pathways [20], Reactome [5, 6] and KEGG [8, 9] databases because of their size and current status as primary sources of data for large-scale models of signal transduction.

To examine the suitability of existing database representations of pathways for computational pathway analysis, we consider five criteria in the areas of knowledge representation and the representation of biological complexity: adherence to standards in implementation, uniqueness of entity references, use of meta-entities to group data, the resolution of captured data, and ability to capture multicellular interactions.

## KNOWLEDGE REPRESENTATION

The accumulated knowledge held in pathway databases is valuable. Good representation enables the development of efficient and accurate analytical techniques, even when working with large multipathway systems or entire databases, whereas poor representation can have the opposite effect. Three key representational issues appear in the current generation of databases: variation in implementation of standards, lack of uniqueness criteria and the use of sets of molecules in interaction and reaction data. Each presents its own challenge to the modelling community.

### DATABASES MAY VARY WIDELY IN IMPLEMENTATION OF STANDARDS

Each database system has a different underlying structure, and captures subtly different information. KEGG, one of the longest-running curation projects, uses DBGet/LinkDB to retrieve records stored in their specialized KEGG Markup Language (KGML) data representation [9]. In contrast, Reactome uses a Structured Query Language (SQL) backend to retrieve data stored in flat files according to the Reactome specification [5]. Even when databases make programmatic access to their data available (through application programming interfaces, or APIs), this makes systematic access to the data contained within the database challenging—any software must be capable of negotiating database-specific features and implementations.

To deal with this problem, data exchange formats such as Systems Biology Mark-up Language (SBML) [21], Biological Pathway Exchange (BioPAX) [22] and the Human Proteome Organisation (HUPO) Protein Standards Initiative Molecule Interaction (PSI-MI) [23] have been developed by the community or different segments thereof. These formats provide common standard methods of data representation that can be accessed programmatically. However, implementation of these standards by each database varies.

Currently, most databases make their data available in several of these formats (Table 1). However, the location of important information (such as entity names or accession numbers for other resources such as UniProt) varies between databases (Supplementary Data S1). This difference in implementation prevents the creation of database-agnostic software, and defeats the purpose of data exchange standards.

**Table 1:** Formats implemented by major databases

Database	BioPAX L3	BioPAX L2	SBML	PSI-MITAB	Custom format	API
Reactome	✓	✓	✓	✓	MySQL dump	✓
PANTHER Pathway	✓	✗	✓	✓	CellDesigner-compatible SBML	✗
KEGG	✗	✗	✗	✗	KGML	✓
NCI-PID	✓	✓	✗	✗	PID XML	✗
PathwayCommons	✓	✗	✗	✗	SIF, tab-delimited	✓

We discuss data sourced from four major databases (Reactome, PANTHER Pathways, KEGG, NCI PID) and a meta-database that aggregates information from multiple sources (Pathway Commons). These databases make their data available both through a graphical web-based interface (with associated diagrammatic representations) and in numerous community-specified and custom formats, as well as through APIs.

Even where standard formats are adopted, the specifications themselves can result in ambiguity (Supplementary Data S1). Standard formats frequently make recommendations and outline best practices, the uptake and implementation of which varies significantly between databases. For example, the BioPAX Level 3 specification provides a description of how to represent complexes, which are physical associations between or among multiple component entities. In this description, it is recommended that a complex should not contain other complexes as their components—instead, they should be ‘flat’ structures containing proteins, small molecules and other participants in the reaction system to prevent implicit encodings of assembly order [24]. This also allows easy checking of components without need for recursion. Because this is described as a recommendation, it is not enforced, and causes further variability in the implementation of the standard.

The solution to this issue would seem to be relatively straightforward—stronger standards for data sharing at the community level, and stricter quality control by database designers. However, any gain in interoperability owing to stricter, more detailed specifications must be carefully weighed against the associated loss of flexibility to capture non-standard information.

### UNIQUENESS CRITERIA ARE NOT GUARANTEED

**Figure 1:**
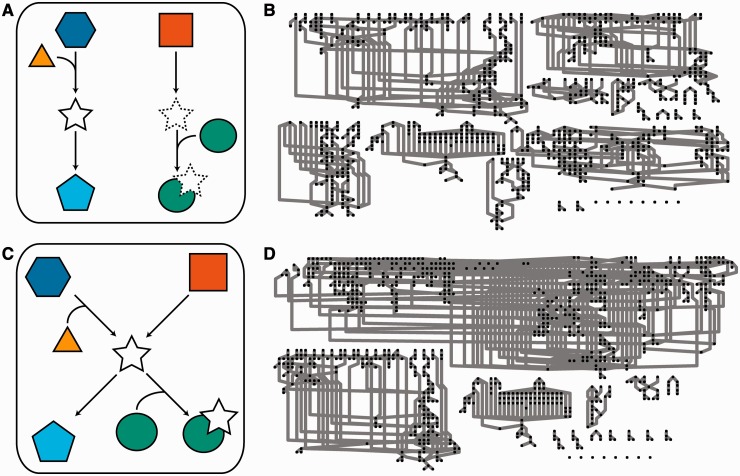
Duplication of entities decreases network connectivity. It is essential that each entity in a given cellular location is represented with a single entry in the underlying database. In this visualization, each node in the network refers to a unique database entry. In (**A**), the entity represented by a star has been duplicated (solid and dashed outlines). This significantly reduces the connectivity and complexity of the network described by the data. (**B**) shows a network consisting of multiple signal transduction pathways implicated in prostate cancer visualized from data originally sourced from the PANTHER Pathways database and analysed in [17]. This network has duplication of 28 entities. Correcting these duplications, as illustrated in (C) yields the network shown in (**D**), with an attendant increase in connectivity and complexity.

Another issue is duplication within the database or model. It is essential that each entity in the system be described by a single unique database entry—that, for example, STAT3 protein in the cytosol with no post-translational modifications (PTMs) is represented uniquely. This is critical from a modelling perspective, and is often assumed. For example, reaction information extracted from the Panther Pathways database was used to form a constraint-based model of signal propagation in multiple signal transduction pathways linked to prostate cancer [17]. However, 28 entities in the system were duplicates (Supplementary Data S1 and S2). Not only did this effectively linearize a connected interdependent network (as demonstrated in Figure 1), the duplication made multiple additional pools of nucleotide triphosphate available for use in the reaction system, which has major implications.

Duplication of entries can cause loss of connectivity within the extracted reaction network (Figure 1) and generate errors in simulation. This loss is severely problematic for topology-based analytical methods, in that it effectively disconnects reactions, arbitrarily and randomly linearizes otherwise complex interconnected relationships and eliminates potentially significant crosstalk between pathways, largely by disconnecting the functions of multifunction proteins. It is important to note that this issue is distinct from the use of duplication in visualizations of the data. Duplications to aid visual interpretation are supported by graphical exchange standards (e.g. [25]) and where they are used, refer to unique entries in the underlying database.

In most interaction databases, duplications are prevented by checking each new entry’s third-party database identifiers, such as UniProt [26] accessions for proteins, ENSEMBL [27] identifiers for genes and unique chemical identifiers such as the IUPAC International Chemical Identifier [28] or Chemical Entities of Biological Interest [29]. These strategies cannot be applied to complexes of entities, or to molecules without third-party database references. Such references can also fall victim to changes in the external databases, such as the deprecation and reassignment of accession numbers [30]. It is important that modellers are aware of the need to confirm that molecules are uniquely and exclusively associated with a single identifier when extracting reaction data from large databases.

### OVERUSE OF META-ENTITIES HAS UNINTENDED CONSEQUENCES

Entity sets are groups of molecules that behave in the same way or fulfil the same role in a reaction, and are therefore interchangeable in that reaction. Generally, these sets operate at a conceptual level as a Boolean ‘OR’ operator. This allows for the capture and description of mechanisms such as catalysis by any member of that group of entities.

**Figure 2:**
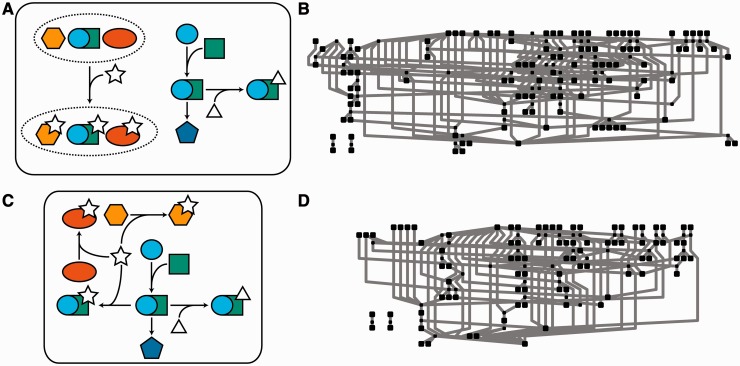
Bucketing of entities has a significant effect on networks. In (**A**), three entities have been grouped into a meta-entity (dashed circle), which interacts with the species described by the star. One of the entities has a number of distinct separate activities outside of this group. The network depicted in (**B**) is sourced from Reactome's ‘Mitotic G1-G1/S phases' pathway (REACT_21267.3). The BioPAX Level 3 representation of this pathway contains 27 of these meta-entities. Removing the meta-entity, as illustrated in (**C**) results in significant changes to the network shape. Restoration of connectivity lost owing to meta-entity use generates the network shown in (**D**), significantly changing network topology.

The widespread use of sets of entities (quantified in Table 2) instead of individuals has major unintended consequences for the modelling and database-curation communities. Some databases permit users to group entities in reactions where the entities undergo similar biochemical modification, with sets of entities as both reactant and product (Figure 2). This creates problems when a member of one of these sets has functions outside of the set. For example, Cyclin A and Cyclin E participate in a number of reactions, some of which require a specific Cyclin, and some of which may use either. Reactome groups the entities Cyclin A (REACT_5498.2) and Cyclin E (REACT_5284.1) into the meta-entity Cyclin E/A (REACT_9091.1) when describing reactions which may use either gene, whereas reactions that require a specific form use the relevant individual identifier. Thus, not all the reactions that can use Cyclin A are associated with the Cyclin A identifier, and shared functions are linked to the Cyclin E/A meta-entity, effectively breaking these connections (as illustrated in Figure 2). The situation becomes more-complex still, as Reactome’s representations of Cyclin A and Cyclin E are themselves meta-entities, as each cyclin has two isoforms [A1 (REACT_4163.1)/A2 (REACT_3467.1) and E1 (REACT_2635.2)/E2 (REACT_2733.2), respectively], and these isoforms have additional distinct reactions from their parent and grandparent meta-entities.

**Table 2:** Frequency of occurrence of meta-entities and non-flat complexes

Database	Total number of entities	Meta-entities	Number of complexes	Number of recursive complexes
Reactome	24 477	2419	6040	3485
KEGG*	25 043	4716	–	–
PANTHER	13 241	Unlabelled^1^	913	34
NCI-PID	27 367	Approx 960^2^	9016	2751

Both meta-entities and non-flat complexes are common in the major signal transduction databases discussed. *KEGG data sourced from their REST API, downloaded as KGML files for interpretation. ^1^Panther Pathways does not annotate their sets of entities–this is only evident from entity names [e.g. ‘C-jun-amino-terminal kinase 1, 2, and 3 (JNK1–3)’ as specified in the ‘FGF signalling pathway’] ^2^NCI-PID does not annotate their sets of entities with a clear marker—an estimate was made by counting the number of entities with cross-references to multiple proteins (e.g. ‘GRP1 family’ from the ‘Arf6 signaling events pathway’).

Meta-entities that bundle entities with distinct function cause issues similar to the duplication of molecules in the database. The severity of these issues are dramatically amplified when they occur in conjunction with complex formation. Complexes are formed by the physical combination and association of their components, and their database representations are conceptually equivalent to Boolean ‘AND’ operators. Complexes may have meta-entities of the type previously discussed amongst their components. These component meta-entities may also contain other subcomponent meta-entities and complexes, adding depth and complexity to the parent complex. One such complex is Cyclin E/A:Cdk2:phospho-p27/p21:SCF(Skp2):Cks1 (REACT_9193.1), which contains two subcomplexes, representing eight possible combinations of its component molecules (Supplementary Data S1). Alternatively, a meta-entity can consist of a set of complexes themselves, which may in turn contain meta-entity components. When such structures and representations occur, the constructions become more than simple sets of equivalent entities, and approach something closer to a grammar.

The repercussions of this structure on the extraction of reaction networks are severe. Essentially, each member of a meta-entity must be checked to determine whether it participates in interactions separate from those of its parent. If it does, then the equivalent molecule must be extracted from the meta-entity representing the product (or reactant) of that interaction. This process must be repeated for all meta-entities containing that molecule or its modified equivalents, until full extraction is achieved. The difficulty here lies not in the decomposition of entity sets or the identification of which members of a given meta-entity have independent function. There are three challenges: (i) the construction of new mass-balanced reactions (a problem discussed in the context of metabolic networks in [31, 32]); (ii) identifying all entities in the network affected by the expansion (as a meta-entity may be a member of another meta-entity or component of a complex); and (iii) editing the original meta-entity from which molecules have been isolated to prevent loss of information regarding other members. This editing step is approximately equivalent to programmatically modifying a complex formal grammar [33] in such a way that it no longer generates a specific sequence, without otherwise modifying its language. In the case of Reactome, where ∼10% of the transducers in the system are meta-entities, ∼2400 new formal grammars may need generation, an onerous and time-consuming task.

Specific databases use different variations of meta-entities. The simplest of these is found in the KEGG database, where molecules with equivalent function are bundled together. An example of this occurs in the ‘FGF’ ligand shown in the MAPK signalling pathway (KEGG PATHWAY:hsa04010), which represents 22 separate molecules acting as agonists of the four receptors bundled under the ‘FGFR’ annotation. A more-complex version is that used by the Reactome database, which features three distinct types of set representation [5, 6]. The first of these, DefinedSet, functions as previously described to group equivalent molecules. The second, CandidateSet, implements a ‘maybe-OR’ functionality used to describe situations in which a group of molecules has been implicated in a process, but the specific molecule involved has not been elucidated. Reactome also provides an ‘OpenSet’ structure, which is a set of molecules with some shared structural property, defined by an example. This classification of set type is not consistently made available in the exported versions of the database (Supplementary Data S1), with the BioPAX version annotating these meta-entities as generic ‘EntitySets’, without providing detail as to which type of set they are. The SBML version of Reactome provides this information for some sets, but also uses the generic ‘EntitySet’ notation for others.

Collecting distinct entities into meta-entities serves two purposes. Firstly, use of meta-entities allows the compression of information that would otherwise cause a database to undergo a combinatorial explosion in size [e.g. Reactome’s ‘APOBEC3G:RTC with deaminated minus sssDNA:tRNA primer:RNA template’ (REACT_9785.1), representing 484 323 840 000 individual molecules (Supplementary Data S1)]. This is a powerful and proper use of these representations, and does not significantly affect the extracted reaction network. However, meta-entities may also be used for ease of data entry and visual representation. This latter use can, as illustrated, cause unintentional loss of connectivity. Ideally, database designs should distinguish between the two, and only the former should be captured in the exported underlying data.

### BIOLOGICAL COMPLEXITY AND CONTEXT

The second area of concern from the perspective of modelling lies in the capture and representation of biological complexity and context. As the largest repositories of pathway reaction data, KEGG and Reactome represent the best starting points for building large-scale models of cellular function. While these databases capture significant portions of the interactome, they do not capture behaviour related to splice variants and protein isoforms, or multicellular interactions, in a manner that enables reaction networks to be easily extracted.

### VARYING LEVELS OF MOLECULAR RESOLUTION

Protein isoforms may be generated by a number of mechanisms (e.g. from homologous genes, by alternative splicing, from post-translational events, etc.). The body of information regarding the activity and effects of different isoforms is steadily growing. Splice variants of proteins are a key source of functional diversity in healthy organisms [34], play major roles in many genetic diseases [35, 36] and have been implicated in cancer [37]. A number of recent articles have specifically implicated splice variation as a mechanism through which signalling pathways vary [38–41]. For example, alternative splicing of the transcript for growth-factor receptor-bound protein 2 (GRB2, UniProt:P62993) leads to production of an isoform, GRB3–3 (UniProt:P62993–2). GRB3–3 is known to have a dominant negative effect over GRB2, triggering apoptosis as part of the programmed cell death pathway [42]. Capturing these behaviours will be important in future modelling efforts.

Additionally, the experimental characterization of protein PTMs (e.g. phosphorylation or ubiquitination) has become more accurate and less expensive over the past decade, with a corresponding increase in the amount of PTM data available. Database editors must deal with and interpret experimental observations of signal transduction events that range in detail from not describing PTMs at all, through those that require some PTMs without regard to specific sites, through to those that require specific combinations of PTMs at specific sites. Some databases attempt to capture information covering protein isoform details and the sites and nature of PTM [5, 7]. At present, the main Reactome database captures a mix of generic [e.g. phospho-Emi1 (UniProt:Q9UKT4, REACT_2668.2)] and site-specific [e.g. p-T161-CDK1 (UniProt:P06493, REACT_5391.4)] PTMs, and some alternatively spliced isoforms are recorded in more recently annotated pathways [e.g. IRAK1 splice variants (associated with UniProt:P51617, REACT_7703.1)]. At an interaction level, this can be handled by the previously described meta-entity structures. It is important that care be taken when working with meta-entity structures owing to the attendant risks of grouping functionally distinct elements, and the subsequent loss of network connectivity.

Failing to capture, or partially capturing, PTMs can result in distortion of the reaction network. For example, there are a number of cases in which a protein must have PTMs at specific sites for a particular interaction to occur. Problems arise when a protein with these modifications at unspecified sites is included within the database, or where an interaction fails to specify these sites. An inclusive approach to these situations risks creating links between subsections of the network that do not exist, whereas a conservative approach that discards such information risks severing links that do.

An additional issue is the problem of curating and maintaining these resources over time. The sequential nature of the curation process means that a given database may have closely related pathways entered years apart (Reactome’s ‘Apoptosis’ pathway dates to 2004 (reviewed February, 2013), and its ‘Cellular Responses to Stress’ pathway to late 2011). A pathway curated in 2013 would almost certainly contain information about isoform-specific function and PTMs that was simply not available 8–10 years ago.

The level of detail captured by reactions in a database can vary widely in terms of this level of detail, even when derived from contemporaneous experimental results reported in the same publication. For example, the previously mentioned phospho-Emi1 (a generically phosphorylated molecule with no site-specificity) has an entry in the ‘Mitotic Metaphase/Anaphase Transition’ pathway (REACT_1016.2). However, another phosphorylated Emi1 with site-specific modifications [phosphor-Emi1(Ser 145, Ser 149), REACT_7080.1] appears in the related ‘APC/C-mediated degradation of cell cycle proteins’ (REACT_6828.1) pathway. The signalling events in which these molecules participate are described in the same publication [43], yet have varying levels of detail in their representation within the database.

This has important implications for the interactions between pathways (or ‘crosstalk’), and requires additional checking against previously grouped sets of molecules. Such crosstalk has been demonstrated to be of key importance in regulation of phenotype [44, 45]. Strategies to deal with this variation in level of detail are required to minimize the impact of variable levels of resolution across individual pathways, and to minimize the resultant distortion of extracted reaction systems. Rather than the current approach of iterative expansion of signal transduction databases (which is the root cause of this divergence), it may be necessary to incorporate a more-thorough revision process specifically targeting differences in resolution at each major release and publication point.

### MULTICELLULAR INTERACTIONS ARE NOT WELL-DESCRIBED

Interactions and reactions described in pathway databases are recorded as occurring between entities that are in a specific subcellular location, such as the cytosol, extracellular region or various organelles. This information is useful in determining the reactions in which a molecule can participate, and captures sequestration and compartmentalization within the cell, in addition to allowing description of events related to secretion and molecular transport.

**Figure 3:**
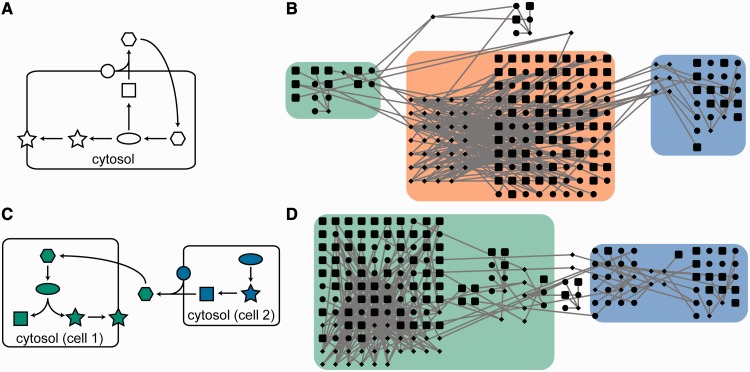
Multicellular interactions present problems in the absence of a defined cellular frame of reference. (**A**) shows an example system with cellular locations defined solely with respect to the cytosol, cell membrane and extracellular region of an unspecified cell. This representation generates ambiguity and is misleading when describing multicellular interactions—the same set of reactions can lead to significantly different functional capabilities of the interaction network when this is accurately represented (**C**). The example in (**B**) is sourced from Reactome’s ‘Latent infection of *H. sapiens* with *M. tuberculosis*’ pathway (REACT_121237.2). In the version of the network described in the database, the ‘cell wall’, ‘periplasmic space’, and ‘plasma membrane’ locations can be assigned to *Mycobacterium* (green) and ‘phagocytic vesicle membrane’ and ‘late endosome membrane’ to *H. sapiens *(blue). The more generic ‘cytosol’ is ambiguous (orange), and reactions assigned to this location could belong to either species. Fixing these assignments (using the graphical representation of the pathway) yields the unambiguous representation shown in (**D**). A colour version of this figure is available at BIB online: http://bib.oxfordjournals.org.

Almost all of the databases mentioned so far also contain information detailing interactions occurring between cells. Recording the cellular location of a given entity becomes more difficult when two (or more) cells interact within the system. For example, the Reactome database contains information governing host–pathogen interactions in latent *Mycobacterium tuberculosis *(tuberculosis) infection. Interactions in this pathway occur in the host cell, in the pathogen or in the extracellular region between the two, and are obviously separated in the pathway visualization. However, no distinction is made between the cytosol of different cells in the exported version (BioPAX Level 3) of the Reactome database (Supplementary Data S1). This results in the effective fusion of the cellular systems of the pathogen and its host, and prevents accurate simulation of the interactions as illustrated in Figure 3. This situation also occurs in cell–cell and synaptic signalling data, where it causes similar problems by collapsing multiple cells into one. Similar problems occur in other databases. Notable examples include the BioPAX version of the NCI-PID curated pathway ‘Effects of *Botulinum* toxin’ inadvertently describing *Botulinum* neurotoxins (e.g. P18640) as human proteins, and the attribution of West Nile Virus RNA to *H. sapiens *in the BioPAX version of the BioCarta-sourced ‘West Nile Virus’ pathway retrieved from the NCI-PID database.

Additional problems arise in the context of whether a reaction can be considered to be part of a normal healthy cell or tissue. Diseases that affect cellular signal transduction are frequently included in databases, but do not distinguish between normal events in healthy cells and events that occur owing to infection. Similarly, databases do not uniformly record information about the tissue specificity of signalling pathways and reactions. This can cause problems with model quality when, for example, synaptic signalling reactions are incorporated into models of inflammation.

One suggestion for avoiding this unintentional merging of organism-, tissue- and disease-specific entities and interactions would be to specify databases at the reaction level with respect to a single canonical cell free of disease, and to support such a model in the specification of data exchange standards. Additional cell types can then be added to provide data relating to additional interacting cells or organisms. A model of an otherwise healthy cell with latent tuberculosis infection would then be represented as the interaction data available for the canonical cell, with the addition of interactions associated with the cell type describing the pathogen. Any contradictions between the two sets of interactions would then have to be resolved. Such an approach would make it easy to generate reaction networks describing both healthy and diseased cells, and even allow generation of clean data sets covering multiple infections (such as HIV-infected cells with latent tuberculosis) for simulation and analysis, a highly desirable outcome for applications to personalized medicine.

## CONCLUSIONS

Databases, including Reactome, KEGG, PID and Panther Pathways, containing broad-scale information represent the best collections of high-quality reaction and interaction data available to the scientific community today. These data sets have immense potential in predictive and explanatory cellular-scale modelling, and enable systems-level descriptions and models of cells.

Features and undocumented issues surrounding the implementations of these databases have important implications for computational network modelling. Databases are designed with multiple stakeholders in mind–the database designers, the curators and data entry teams, small-scale analysts, large-scale analysts and people using visual analysis of pathway maps, all have different requirements, and some of the issues we discuss here are a product of compromise among the competing demands of these stakeholders [46]. Our analysis is based on the exported exchange files (e.g. BioPAX and SBML formatted data), which are the most common, and in the case of Panther and PID, the only method for accessing the databases’ content. The majority of problems we describe also occur in the underlying databases (the notable exception being Reactome’s clear labelling of interactions with the species in which they occur in their SQL database, although not in their exported BioPAX files), which makes resolution of these problems difficult.

Our analysis has focused on signal transduction systems; however, databases describing these and other processes in a systems context face similar challenges. A good example is that of metabolism, where the primary modelling techniques require mass- and stoichiometrically-balanced reactions. Grouping of entities through reactions at the database level makes instantiating models based on these data vastly more difficult. Addressing these issues for metabolic systems has required extensive reworking and modification of databases to ensure that they can be used for and facilitate modelling applications [31].

The advent of whole-cell computational modelling and the expanding use of high-throughput techniques require ever more-sophisticated data analysis methods. While established first-generation methods such as enrichment analysis do not suffer greatly from the issues described here, the structural issues that we discuss become limiting and will be addressed as the field moves beyond enrichment and towards mechanistic models. Handling these issues and thus facilitating systematic extraction of basic reaction information will allow development and improvement of computational data analysis using these data sets. This combination will drive new and exciting experimental work, and dramatically enhance our ability to work with and exploit cellular level phenomena in a systematic, evidence-based fashion.

## SUPPLEMENTARY DATA

Supplementary data are available online at http://bib.oxfordjournals.org/.

**Key Points**

- Large-scale models using prior knowledge about detailed signal transduction mechanisms are becoming more common.
- These models require the extraction of a reaction network, and one source of such data is community-sourced data in major databases.
- Knowledge representation in these databases has unintended consequences for computational modelling.
- Use of sets of entities and duplication of entities cause unintended loss of connectivity in database-derived reaction networks.
- Multicellular interactions are not currently well captured in databases and can result in misleading reaction networks.
- Additional work is needed to capture behaviours such as splice variation of proteins in a way that does not further hinder analyses.

Supplementary Data
