# Supplementary material for: Extracting reaction networks from databases–opening Pandora’s box
Source: Brief Bioinform. 2013 Aug 14;15(6):973–83. doi: 10.1093/bib/bbt058 (PMC4239801; doi:10.1093/bib/bbt058)
Supplement: Supplementary Data [file supp_15_6_973__index.html]

Extracting reaction networks from databases–opening Pandora’s box — Extracting reaction networks from databases–opening Pandora’s box — Supplementary Data 

# Extracting reaction networks from databases–opening Pandora’s box

## Supplementary Data

files

**Files in this Data Supplement:**

- Supplementary Data - pdf file
- Supplementary Data - xls file
