# Supplementary material for: Extracting reaction networks from databases–opening Pandora’s box
Source: Brief Bioinform. 2013 Aug 14;15(6):973–83. doi: 10.1093/bib/bbt058 (PMC4239801; doi:10.1093/bib/bbt058)
Supplement: Supplementary Data [file supp_bbt058_Fearnley_et_al-Supplementary_S1.pdf]

Supplementary Information S1 : Extracting Reaction Networks from Databases – Opening Pandora's Box

Fearnley, L.G., Davis, M.J., Ragan, M.A., Nielsen, L.K.

1 Data Sources and Versions

1.1 Reactome

We discuss data sourced from the Reactome [7] download site (`http://www.reactome.org/download/index.html`) at 9.08AM 8/11/2012, AEST. Interactions and entities discussed in this work are described in the BioPAX Level 3 and SBML (level 2, version 3) formats made available on that page.

1.2 NCI-Nature Pathway Information Database (PID)

We discuss data sourced from the NCI-Nature PID [9] download site `http://pid.nci.nih.gov/download.shtml` at 9.15AM 8/11/2012, AEST.[1] Interactions and entities discussed in this work are described in the 'NCI-PID Curated' and 'BioCarta' data sets, both in BioPAX Level 3 format.

1.3 PANTHER Pathways

We discuss data sourced from the PANTHER Pathways [10] download FTP server `ftp://ftp.pantherdb.org/pathway/3.1/` at 9.33AM 8/11/2012, AEST - version 3.1 of the database. PANTHER Pathways data is curated using the CellDesigner software [4] and exported from this into BioPAX.

1.4 Kyoto Encyclopædia of Genes and Genomes

Data from KEGG [5] was sourced from the KEGG PATHWAY database between March and September 2012. KEGG offers two methods of access to its underlying data - subscription based access via FTP to the full dataset, or access to descriptions of individual pathways via download links on the graphical representation of each pathway or via the get method of its API. We have included examples from KEGG to illustrate the applicability of our points to multiple databases, but due to the lack of access to the entire dataset, have not used KEGG as a primary resource.

[1] NCI-PID was a database project resulting from a collaboration between the National Cancer Institute (US) and Nature Publishing Group. This collaboration was active from 2006 through to September 2012, and is now dormant - the data will continue to be hosted at this address until September 2013.

2 Variation in Implementation

**Data:**	PANTHER Pathways:	'JAK_STAT_signalling_pathway.owl'
	Reactome:	'Homo sapiens.owl'
	NCI-PID:	'NCI-Nature_Curated.bp3.owl'

Note: Data entry and curation in the PANTHER Pathways database is handled using the CellDesigner software, which provides graphical editing of interaction networks. This data can then be exported to the various levels of the BioPAX format [1] from this software. As such, comments about structure and format implementation of PANTHER's data are generalisable to *all* databses specified with this software.

2.1 Storing Data

One major problem from an analytical perspective is variation in where databases store information about participants in the reactions they describe. This variation can happen even when the databases use identical formats to store their information.

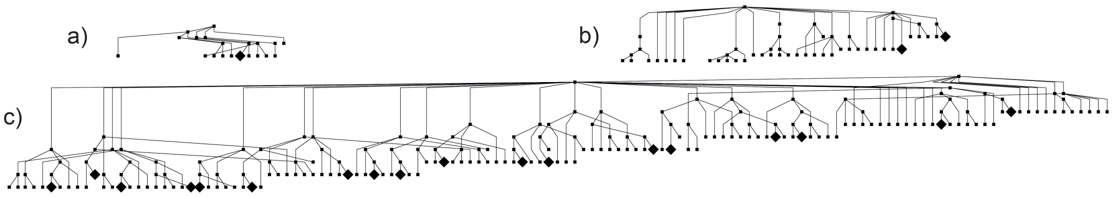

Figure 1: Three databases (NCI-PID, PANTHER, and Reactome) describe post-translational modification of the JAK protein (UniProt:P23458). Each database varies in terms of how and where they store the Uniprot accession data (larger diamonds) for this protein. We display a tree-based representation of these records where the root is the parent record, each internal node represents a field or sub-field in the database, and the leaf nodes represent values. a) NCI PID representation. b) Reactome representation. Note two UniProt accessions are stored - both containing the same accession details in different formats ('P23458' and 'UniProt:P23458 JAK1'). c) PANTHER Pathways representation records multiple UniProt accession details, including those for orthologs in other species, and a number of alternative accessions. 'P23458' is one of many captured.

For example, most databases cover the post-translational modifications of Janus kinase (JAK) (UniProt:P23458). We have selected three records describing JAK phosphoproteins with two features (non-identical due to lack of overlap) from PANTHER Pathways, Reactome and NCI-PID to illustrate this variation. These are shown in diagrammatic form in Figure 1 and in a detailed tabular representation in the appendix to this document (Tables 4, 5, & 6).

In the worst case situation, this variation necessitates a complete search of every attribute linked to a given biochemical entity (complex, protein, small molecule, RNA

or DNA) in a reaction system to find a given attribute. If these attributes need to be frequently accessed, as they are during the mapping of experimental data labelled with UniProt [11] or ENSEMBL [3] identifiers to computational models, then the number of searches required become problematic.

Other variation arises from ambiguity within the specification itself. For example, the description of the implementation of Complexes in the BioPAX Level 3 specification (pg. 48 [1]) states the following:

> In general, complexes should not be defined recursively so that smaller complexes exist within larger complexes, i.e. a complex should not be a component of another complex (to avoid errors in interpretation - see comments on the component property below). Instead, the subunits should be a simple list.
>
> **component** - (0 or more object:PhysicalEntity) Defines the PhysicalEntity subunits of this complex. This property should not contain other complexes, i.e. it should always be a flat representation of the complex. For example, if two protein complexes join to form a single larger complex via a complex assembly interaction, the component of the new complex should be the individual proteins of the smaller complexes, not the two smaller complexes themselves.

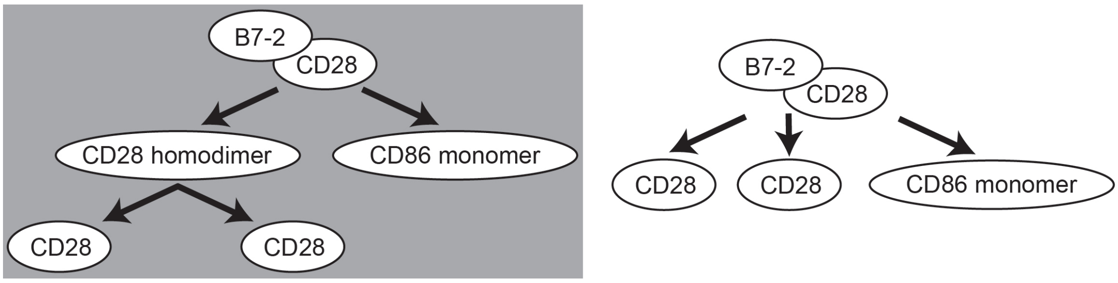

Figure 2: a) Recursive complexes can be multi-level and encode implicit assembly information. Determining membership or participation in a complex requires recursion through all elements of a complex, adding additional overhead in very large models. b) Flat complex representations are recommended in the BioPAX Level 3 specification, and prevent many of the problems outlined here.

This recommendation not implemented by any of the major databases. The NCI-PID data contains 2,751 (of 9,016) recursively defined complexes, Reactome, 3,485 (of 6,040), and Panther 34 (of 913). This is problematic for a number of reasons. Firstly the presence of recursively defined complexes implies an assembly order that is not necessarily biologically or mechanistically correct. Secondly, mapping components becomes difficult due to the need to recurse to the bottom of the resulting 'Complex tree' that results from this type of structure. Finally, this makes checking to see whether a Complex is duplicated vastly more difficult (Figure 2).

3 Uniqueness Criteria

Data: Dasika et al [2] Supplemental Data [2]
Reactome: 'Homo sapiens.owl'

Entries in a database should be unique — if the database describes adenosine-5'-triphosphate (ATP, CAS:56-65-5) in the cytosol of a cell, this should be represented by a single entry for cytosolic ATP. Duplication of entries leads to loss of network connectivity - the participations of the molecule are fragmented and no longer connected.

This poses a major problem for modelling applications due to the importance of capturing crosstalk, or interactions between pathways. In current pathway databases, data is curated in a functional fashion — a team of curators begin looking at some functional process (for example, apoptosis), and conduct extensive searches of the literature to enumerate the set of interactions and reactions that have been determined to be relevant to that process [7, 9, 5]. Because of this, the main points at which crosstalk can occur lie at the intersections of the sets of participants in individual curated pathways. Duplication of entities in the network obscures this overlap, and prevents capture of signal flow.

Examples of unexpected duplication are common in the modelling literature. For example, Dasika et al [2] describe a modelling experiment where nine signalling pathways (sourced from the PANTHER Pathways database) implicated in prostate cancer are combined for simulation using constraint-based techniques. They provide their data as supplementary material. This data has a large number of duplications, with significant effect on the topology of the interaction network (Figure 1 in main paper, table describing duplication as supplementary file).

This problem is not constrained to the PANTHER Pathways data. Precisely determining the level of duplication in a given database is difficult. A given entry in the database contains a set of names, a cellular location, a set of identifiers (internal and external), and a set of modifications and features (e.g. posttranslational modifications in proteins). All of these properties (with the exception of the databases' internal identifiers, which are unique and set at data entry) need to be considered when comparing entries for identification purposes.

[2] `http://dx.doi.org/10.1529/biophysj.105.069724`

4 Meta-entity examples

**Data:**	Reactome:	'Homo sapiens.owl'
	KEGG	'MAPK Signaling Pathway' (hsa04010) [3]

Bucketing of entities is described in detail in the main text of the work. We begin by discussing the provided examples in more detail:

4.1 Reactome : Cyclin E/A:Cdk2:phospho-p27/p21:SCF(Skp2):Cks1 (REACT_9193.1)

This entity is involved in the regulation of the cell cycle — cyclin E/A complexed with CDK2 is a key checkpoint kinase involved in mediating the transition from G1 to S phase. p27 or p21 inhibit the checkpoint kinase through complexation and subsequent p27/p21 phosphorylation. Further complexation with SCF(Skp2):Cks1 complex can then occur, leading to the ubiquitination and subsequent degredation of the molecule.

This entry in Reactome represents 8 possible molecules. They are shown below:

(SCF(Skp2):Cks1):phospho-p21(Ser 130):Cdk2:Cyclin A1
(SCF(Skp2):Cks1):phospho-p21(Ser 130):Cdk2:Cyclin A2
(SCF(Skp2):Cks1):phospho-p27(Thr 187):Cdk2:Cyclin A1
(SCF(Skp2):Cks1):phospho-p27(Thr 187):Cdk2:Cyclin A2
(SCF(Skp2):Cks1):phospho-p21(Ser 130):Cdk2:G1/S-specific cyclin E1
(SCF(Skp2):Cks1):phospho-p21(Ser 130):Cdk2:G1/S-Specific cyclin E2
(SCF(Skp2):Cks1):phospho-p27(Thr 187):Cdk2:G1/S-specific cyclin E1
(SCF(Skp2):Cks1):phospho-p27(Thr 187):Cdk2:G1/S-Specific cyclin E2

Table 1: Individual components of metaentity 'Cyclin E/A:Cdk2:phospho-p27/p21:SCF(Skp2):Cks1' in the Reactome *Homo sapiens* signal transduction dataset.

[3] `http://dx.doi.org/10.1529/biophysj.105.069724`

4.2 KEGG: FGF

KEGG currently offers subscription-only access to their underlying data. The publically available data is presented as sets of pathway diagrams. One of these, the diagram describing the 'MAPK signalling pathway' (hsa04010)[4], contains 46 bucketed entities and 13 unique generic events (such as 'Proliferation, differentiation').

4.3 Reactome : APOBEC3G:RTC with deaminated minus sssDNA:tRNA primer:RNA template (REACT_9785.1)

This entity is described in the HIV-host interactions section of the Reactome database. It is a terminal node in the network, and represents the outcome of APOBEC3G-mediated minus-strand deamination over the HIV genome.

This entry in Reactome represents 484,323,840,000 possible molecules. A tabular description (similar to that used in the prior subsection) is shown below:

[4] http://www.genome.jp/dbget-bin/www_bget?pathway:hsa04010

APOBEC3G:RTC with deaminated minus sssDNA:tRNA primer:RNA template [cytosol]
APOBEC3G [cytosol] - 2
RTC with minus sssDNA containing deaminated C residues:tRNA primer:RNA template [cytosol] - 242,161,920,000
2 x Integrase [cytosol] - 40 (20 options x2)
Rev [cytosol] - 30
Vif [cytosol] - 22
Vpr protein [cytosol] - 20
Vpu protein [cytosol] - 26
p6 protein [cytosol] - 21
RT [cytosol] - 40
p66 subunit of RT [cytosol] - 20
p51 subunit of RT [cytosol] - 20
Matrix [cytosol] - 21
CypA protein [cytosol] - 1
minus sssDNA containing deaminated C residues:RNA template:tRNA primer [cytosol] - 1
HIV-1 RNA template [cytosol] E - 1
tRNA-Lysine3 [cytosol] - 1
minus sssDNA containing deaminated C residues [cytosol] - 1

Table 2: Individual components of metaentity 'APOBEC3G:RTC with deaminated minus sssDNA:tRNA primer:RNA template [cytosol]' in the Reactome *Homo sapiens* signal transduction dataset, with a total of 484,323,840,000 possible molecules.

5 Multicellular Systems and Cell-Cell Interaction

Many databases describe interaction systems across multiple cells, such as paracrine signalling, synaptic signalling and host-pathogen interactions. Problems arise when these databases use only subcellular localisation. An example of this is in the BioPAX Level 3 version of the Reactome 'Latent infection of *Homo sapiens* by *Mycobacterium tuberculosis*.' pathway. This pathway describes the internalisation of *Mycobacterium* by macrophages, and the countering of the innate immune response prior to entering its persistent state [8, 6].

The diagrammatic representation of the pathway[5] shows two unlabelled cells interacting, and is suggestive of a two-part interaction system. In contrast, the BioPAX version of the same pathway makes no distinction between the subcellular locations of the two cells — ie, the cytosol of the bacterium and the cytosol of the host cell are merged. This merging of bacterial and human reactions and interactions dramatically changes the interaction system and its capabilities, introducing significant error in models derived from these data.

**Location**	**Original**	**Fixed**
cytosol	122	-
H.s. cytosol	-	113
M.t. cytosol	-	17
phagocytic vesicle lumen	12	12
phagocytic vesicle membrane	20	20
periplasmic space	8	8
plasma membrane	17	17
late endosome membrane	1	1
cell wall	3	3
extracellular region	6	6

Table 3: Entities participating in the Reactome pathway 'Latent infection of *Homo sapiens* by *Mycobacterium tuberculosis*' by subcellular location. The 'Original' column contains counts for the pathway as available from the Reactome database, whereas the 'Fixed' column contains the counts for the pathway derived from this with assignment to species-specific subcellular locations.

References

[1] BioPAX Consortium, *BioPAX: Biological pathways exchange.*, 2006, Available online at http://www.biopax.org. Retrieved June 2011.

[5] `http://dx.doi.org/10.3180/REACT_121237.1`

[2] Madhukar S. Dasika, Anthony Burgard, and Costas D. Maranas, *A computational framework for the topological analysis and targeted disruption of signal transduction networks.*, Biophysical Journal **91** (2006), no. 1, 382–398 (eng).

[3] Paul Flicek, M Ridwan Amode, Daniel Barrell, Kathryn Beal, Simon Brent, Denise Carvalho-Silva, Peter Clapham, Guy Coates, Susan Fairley, Stephen Fitzgerald, Laurent Gil, Leo Gordon, Maurice Hendrix, Thibaut Hourlier, Nathan Johnson, Andreas K. Kahari, Damian Keefe, Stephen Keenan, Rhoda Kinsella, Monika Komorowska, Gautier Koscielny, Eugene Kulesha, Pontus Larsson, Ian Longden, William McLaren, Matthieu Muffato, Bert Overduin, Miguel Pignatelli, Bethan Pritchard, Harpreet Singh Riat, Graham R S. Ritchie, Magali Ruffier, Michael Schuster, Daniel Sobral, Y Amy Tang, Kieron Taylor, Stephen Trevanion, Jana Vandrovcova, Simon White, Mark Wilson, Steven P. Wilder, Bronwen L. Aken, Ewan Birney, Fiona Cunningham, Ian Dunham, Richard Durbin, Xose M. Fernandez-Suarez, Jennifer Harrow, Javier Herrero, Tim J P. Hubbard, Anne Parker, Glenn Proctor, Giulietta Spudich, Jan Vogel, Andy Yates, Amonida Zadissa, and Stephen M J. Searle, *Ensembl 2012.*, Nucleic Acids Research **40** (2012), no. Database issue, D84–D90 (eng).

[4] A. Funahashi, N. Tanimura, M. Morohashi, and H. Kitano, *Celldesigner 3.5: A versatile modeling tool for biochemical networks*, Proceedings of the IEEE **96** (2008), no. 8, 1254–1265.

[5] M. Kanehisa and S. Goto, *KEGG: Kyoto Encyclopedia of Genes and Genomes.*, Nucleic Acids Research **28** (2000), no. 1, 27–30 (eng).

[6] Judith Clancy Keen and Nancy E Davidson, *The biology of breast carcinoma.*, Cancer **97** (2003), no. 3 Suppl, 825–833 (eng).

[7] L. Matthews, G. Gopinath, M. Gillespie, M. Caudy, D. Croft, B. de Bono, P. Garapati, J. Hemish, H. Hermjakob, B. Jassal, A. Kanapin, S. Lewis, S. Mahajan, B. May, E. Schmidt, I. Vastrik, G. Wu, E. Birney, L. Stein, and P. D'Eustachio, *Reactome knowledgebase of biological pathways and processes.*, Nucleic Acids Research **37** (2008), D619–22, PMID: 18981052.

[8] D.G. Russell, C.E Barry, 3rd, and J.L. Flynn, *Tuberculosis: what we don't know can, and does, hurt us.*, Science **328** (2010), no. 5980, 852–856 (eng).

[9] Carl F Schaefer, Kira Anthony, Shiva Krupa, Jeffrey Buchoff, Matthew Day, Timo Hannay, and Kenneth H Buetow, *PID: the Pathway Interaction Database*, Nucleic Acids Research **37** (2009), no. Database issue, D674–D679 (eng).

[10] P.D. Thomas, A. Kejariwal, M.J. Campbell, H. Mi, K. Diemer, N. Guo, I. Ladunga, B. Ulitsky-Lazareva, A. Muruganujan, S. Rabkin, J.A. Vandergriff, and O. Doremieux., *PANTHER: A browsable database of gene products organized by biological function, using curated protein family and subfamily classification*, Nucleic Acids Research **31** (2003), 334–341.

[11] UniProt Consortium, *Reorganizing the protein space at the universal protein resource (UniProt).*, Nucleic Acids Research **40** (2012), no. Database issue, D71–D75 (eng).

Table 4: PANTHER Pathways representation.

Protein	_Jak_s10_sa9_					
	displayName	Jak				
	standardName	Jak				
	comment	SPECIES_TYPE=PROTEIN				
		ACTIVATION=TRUE				
	cellularLocation	_Cytoplasm_c2_				
		term	Cytoplasm			
		xref	GO:0005737			
			name	GO:0005737		
			db	GO		
	feature	_Jak_s10_sa9_rs1_				
		comment (×4)	MODIFICATION_RESIDUE_SIDE=none			
		modificationType	MODIFICATION_FEATURE_phosphorylated			
			term	phosphorylated residue		
			comment	DATA_SOURCE=CellDesigner		
			xref	MOD:00696		
				id	MOD:00696	
				db	MOD	
	feature	_Jak_s10_sa9_Active_				
	entityReference	_Jak_PROTEIN_				
		displayName	Jak			
		name	Janus kinase			
			Tyk2			
		comment(3)	ENTITY_REFERENCE_NOTES=Long Name: Jak...Accession:P01034			
		memberEntityReference*	_Jak_HUMAN%7CENSEMBL%3DENSG00000162434%7CUniProtKB%3DP23458			
			name	Tyrosine-protein kinase JAK		
			organism	HUMAN		
				name	Homo sapiens	
				xref	NEWT:9606	
					id	9606
					db	NEWT
			xref	UniProtKB_P23458		
				id	P23458	
				db	UniProtKB	

*1/19 memberEntityReferences displayed due to space constraints. memberEntityReference containing P23458 shown.

Table 5: Reactome representation

Protein	Protein2770				
	displayName	p-JAK1			
	name (\times 3)	Phosphorylated tyrosine-protein kinase JAK1			
	feature	ModificationFeature646			
		modificationType	SequenceModificationVocabulary60		
			term	(2S,4R)-4-hydroxyproline	
			xref	UnificationXref45216	
				id	MOD:00039
				db	MOD
	feature	FragmentFeature1505			
		featureLocation	SequenceInterval1533		
			sequenceIntervalBegin	SequenceSite3645 (sequencePosition:1, positionStatus:EQUAL)	
			sequenceIntervalEnd	SequenceSite3646 (sequencePosition:1154, positionStatus:EQUAL)	
	comment	Reactome DB_ID: 451921			
	cellularLocation	CellularLocationVocabulary4			
		term	cytosol		
		xref	UnificationXref59999		
			db	Reactome	
			id	REACT_123719	
			idversion	1	
			comment	Reactome stable identifier...	
	entityReference	ProteinReference1086			
		name (4)	UniProt:P23458 JAK1		
		comment	FUNCTION Tyrosine kinase of the non-receptor type...		
		organism	BioSource1		
			name	Homo sapiens	
			xref	UnificationXref2	
				db	NCBI_taxonomy
				id	9606
		xref	UnificationXref8070		
			db	UniProt	
			id	P23458	
	xref (×2, Reactome)	UnificationXref11413			
		db	Reactome		
		id	REACT_24350		
		idVersion	1		
		comment	Reactome		
	dataSource	Provenance1			
		name	Reactome		
		comment	http://www.reactome.org		

Table 6: NCI PID representation.

Protein	pid_152243				
	name	JAK1			
	feature	pid_32990			
		type:	pid_174 (name:residue modification, active)		
	feature	pid_32991			
		type:	pid_704		
			db:	PSI-MOD	
			id:	MOD:00048	
			name:	O4'-phospho-L-tyrosine	
	entityReference	pid_16755			
		name:	JAK1		
		xref:	pid_16756		
			db:	UniProt	
			id:	P23458	
		organism:	pid_1		
			standardName	Homo sapiens	
			xref	pid_2	
				db	Taxonomy
				id	9606
